# Effect of adjusting the combination of budesonide/formoterol on the alleviation of asthma symptoms

**DOI:** 10.1186/s40733-018-0043-8

**Published:** 2018-05-21

**Authors:** Ryosuke Souma, Kumiya Sugiyama, Hiroyuki Masuda, Hajime Arifuku, Kentaro Nakano, Hiroyoshi Watanabe, Tomoshige Wakayama, Shingo Tokita, Masamitsu Tatewaki, Hideyuki Satoh, Kenya Koyama, Yumeko Hayashi, Fumiya Fukushima, Hirokuni Hirata, Masafumi Arima, Kazuhiro Kurasawa, Takeshi Fukuda, Yasutsugu Fukushima

**Affiliations:** 1 Department of Respiratory Medicine and Clinical Immunology, Dokkyo Medical University, Saitama Medical Center, 2-1-50 Minami-koshigaya, Koshigaya, Saitama 343-8555 Japan; 2 Department of Pulmonary Medicine and Clinical Immunology, Dokkyo Medical University, 880 Kita-kobayashi, Mibu, Tochigi 321-0293 Japan; 3 Department of Rheumatology, Dokkyo Medical University, 880 Kita-kobayashi, Mibu, Tochigi 321-0293 Japan

**Keywords:** Adjustable maintainable dose (AMD), Asthma, Budesonide, Formoterol, Peak expiratory flow (PEF), Symbicort maintenance and reliever therapy (SMART)

## Abstract

**Background:**

The combination of budesonide + formoterol (BFC) offers the advantages of dose adjustment in a single inhaler according to asthma symptoms. We analyzed the relationship between asthma symptoms in terms of peak expiratory flow (PEF) and dose adjustment by the patient.

**Methods:**

Twenty-eight patients with asthma who used BFC for alleviation of their symptoms (12 men, 16 women; 60 years old) were instructed that the inhaled BFC dose could be increased to a maximum of 8 inhalations per day according to symptom severity. Patients measured and recorded PEF every morning and evening in their asthma diary along with their symptoms and the dose of drugs taken.

**Results:**

Sixteen of the 28 patients increased their dose for asthma symptoms. The time to recovery from the asthma symptoms was significantly shorter when cough was the only symptom present compared with dyspnea or wheeze (1.4 vs. 5.3 or 6.6 days, *p* < 0.05) and when they had only one symptom compared with two or three symptoms (1.3 vs. 5.7 or 10.5, *p* < 0.01). The relationship between PEF (% of personal best) when the dose was increased (Y) and the days for the increased dose to achieve a PEF greater than PEF in the symptom-free state (X) was determined to be Y = − 0.591X + 89.2 (r^2^ = 0.299, *p* < 0.001).

**Conclusion:**

As a guide for increasing the BFC dose when patients with mild asthma have asthma symptoms, the dose should be increased when cough is present or PEF is decreased to 88.9% (i.e., X = 0.5).

## Background

One of the advantages of using the combination of budesonide + formoterol (BFC; Symbicort®, Turbuhaler®, AstraZeneca, Osaka, Japan) is that the dose can be adjusted in a single inhaler according to the severity of asthma symptoms [1–3]. The dose of BFC can be increased during its regular usage times, such as morning and/or evening, and taken as the adjustable maintainable dose (AMD). Also, patients can use additional BFC as needed when they have asthma symptoms. The abovementioned therapy is referred to as Symbicort® maintenance and reliever therapy (SMART), and could reduce the risk and rate of severe asthma exacerbations in patients with moderate to severe asthma [4]. SMART contributes to solving the problems associated with poor adherence to the maintenance dose of inhaled corticosteroids and overuse of short-acting muscarinic antagonists [5]. In the COMPASS study, SMART achieved a significantly greater reduction in the severity of exacerbations of asthma compared with fixed BFC or salmeterol + fluticasone [6]. In the COSMOS study, SMART was more cost-effective and showed a significantly greater reduction in the number of cases with asthma exacerbations compared with salmeterol+fluticasone and short-acting β2-agonist (SABA) [7, 8]. Subgroup analysis of the data obtained in the COSMOS study revealed similar results in Asian patients [9]. In adolescent patients with asthma, SMART showed efficacy and safety consistent with that reported for adults [10]. For patients with mild asthma, the phase 3 trials of the SYmbicort® Given as needed in Mild Asthma (SYGMA) program is currently underway [11]. The results of many other studies are in favor of the use of SMART for controlling the severity of asthma [12–16]. However, studies have also shown that sputum and biopsy eosinophil counts were higher with the use of SMART compared with the use of a high fixed dose of BFC, although this difference in eosinophil counts has no detrimental effect on the control of asthma [17].

As stated above, many studies support the finding that SMART reduces exacerbations of asthma and prevents overuse and/or single-use of SABA and/or long-acting beta-agonists, although some do not. A key aspect of the advantages and disadvantages of SMART is the timing of BFC dose adjustment, because most previous studies on SMART have no detailed description of the timing of increasing the BFC dose, simply referring to the timing as “as needed”. We anticipate that the key to explaining these conflicting results is to determine the conditions under which the BFC dose should be increased. In this study, we analyzed the relationship between various parameters, including peak expiratory flow (PEF) and symptoms, and the time point at which the BFC dose was adjusted by the patient to determine the optimal management of asthma symptoms with BFC. Evidence supporting the use of SMART may be further strengthened if the timing of increasing the BFC dose is explained to patients in detail, based on our results.

## Methods

### Study design

We enrolled patients with asthma who used BFC for alleviation of their asthma symptoms over a period of 4 weeks, as defined in the Global Strategy for Asthma Management and Prevention, in whom daytime symptoms, limitation of activities, and nocturnal symptoms/awaking were all absent, indicating that the asthma control test (ACT) scores were 25 points [18, 19]. We excluded patients with chronic respiratory disease or other chronic diseases associated with cough, such as gastroesophageal reflux disease and sinusitis. We explained to the patients that they could increase the BFC dose (160 μg budesonide and 4.5 μg formoterol per dose) to a maximum of 8 inhalations per day, depending on the severity of their asthma symptoms. For adjusting the dose of BFC, SMART was permitted only for alleviation of asthma symptoms, and AMD was permitted in cases where patients identified their asthmatic symptoms after measuring their PEF in the morning or evening. We also explained to the patients that they were to continue the increased dose of BFC while symptoms were present, and that they were to decrease the BFC dose to the original dose when asthma symptoms were consistently absent. Patients measured PEF every morning and evening and recorded their PEFs in their asthma diary as well as their symptoms and the dose of drugs taken. We then analyzed the relationship between the time when the dose of BFC was increased and their PEF and symptoms. They were also allowed to receive treatment in the emergency department asthma symptoms were not relieved by AMD or SMART. However, no patients were treated in the emergency department.

The prospective study was approved by the ethics committee of Dokkyo Medical University Hospital (No. 22072) and the ethics committee of Dokkyo Medical University Koshigaya Hospital (No. 1409). This study was registered at the University Hospital Medical Information Network Center, Clinical Trials Registry (UMIN-CTR: No. UMIN000009599). Written informed consent for participation in the study was obtained from all patients.

### Patients

Thirty-three patients were enrolled in this study, but 5 patients were later excluded (1 withdrew, 1 did not use BFC regularly, 1 had some asthma symptoms every day, and 2 did not measure PEF regularly), leaving data from the remaining 28 patients for analysis. The baseline characteristics of the patients are shown in Table 1. Sixteen patients adjusted the dose of BFC according to the severity of their asthma symptoms. However, the other 12 used BFC at a fixed dose, although we advised them on the adjustment of BFC, according to their asthma symptoms. There was a significant difference in sex between the fixed-dose and adjusted-dose groups (*p* < 0.05). No significant differences were observed in other baseline characteristics. Patients using both AMD and SMART regimens were 4 women (mean age: 60.8 years), and 12 patients increased their dose of BFC by AMD. Additional number of inhalations was 2.0 ± 0.5 inhalations per day and mean time to decrease the BFC dose was 2.6 ± 5.3 days per event when patients increased the BFC dose.

**Table 1:** Patient baseline characteristics

	Total	Fixed-dose group	Adjusted-dose group
Patients (n)	28	12	16
Mean age (years)	59.7 ± 14.6	65.5 ± 9.8	55.3 ± 16.3
Male/female*	12/16	8/4	4/12
Duration (years)	9.6 ± 7.3	12.5 ± 8.1	7.5 ± 6.0
Atopy/non-atopy	10/18	4/8	6/10
Smoking:			
Smoker	2	1	1
Ex-smoker	4	2	2
Never-smoker	22	9	13
Serum IgE (IU/mL)	420 ± 404	382 ± 287	459 ± 514
Serum eosinophils (/μL)	457 ± 331	537 ± 354	394 ± 308
Pulmonary function:			
FVC (% of pred.)	103.2 ± 12.8	96.6 ± 12.1	107.6 ± 11.6
FEV_1_ (% of pred.)	84.4 ± 21.5	76.1 ± 28.3	90.0 ± 13.8
FEV_1%_	65.5 ± 12.9	60.3 ± 15.7	68.9 ± 9.7
V_50_ (% of pred.)	47.3 ± 26.2	40.5 ± 28.7	51.8 ± 24.4
V_25_ (% of pred.)	30.6 ± 17.2	28.9 ± 16.4	31.8 ± 18.1
Concomitant drugs:			
BFC alone	12	4	8
with LTRA^a^	13	6	7
with theophylline	9	5	4
This study:			
Observation period (days)	253 ± 59	229 ± 74	271 ± 37
Frequency of PEF (%)	94.9 ± 9.6	92.4 ± 14.2	96.7 ± 3.2
Basal dose of BFC (inhalations/day)	3.2 ± 1.0	3.7 ± 0.8	2.9 ± 1.0
Additional dose of BFC** (inhalations/day)	1.0 ± 1.1	0.0 ± 0.0	2.0 ± 0.5

Mean ± SD

*p < 0.05, **p < 0.01 for comparison between the fixed-dose and adjusted-dose groups

^a^Leukotriene receptor antagonist

### Statistical analysis

The personal best PEFR in the observation period was set as 100%. All statistical analysis was performed using Microsoft Excel® 2007 SP3 MSO (Microsoft Corp., Redmond, WA) and JMP® Pro version 11.2.0 (SAS institute, Cary, NC) statistical software. Differences between two independent samples were examined by the chi-square test, Mann-Whitney U test. Differences between two related samples were examined by Wilcoxon signed-rank test. Relationships between two parameters were examined by correlation coefficients and regression line analysis. Differences at *p* < 0.05 were considered significant. The results are expressed as mean ± standard deviation (SD).

## Results

### Difference between the fixed-dose and adjusted-dose groups in terms of PEF changes

**Fig. 1:**
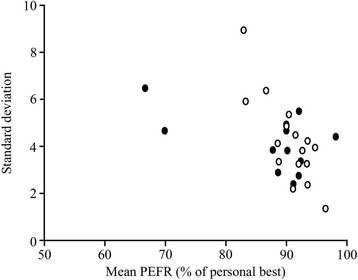
Difference in PEF fluctuation between the BFC fixed-dose and adjusted-dose groups. There was no significant difference in PEF changes between the fixed-dose group (closed circles) and the adjusted dose group (open circles)

PEF changes are shown in Table 2. We evaluated the PEF changes and calculated the mean and SD for each patient, as well as the coefficient of variation (CV: standard deviation/mean) (Table 2). Also, Fig. 1 shows the relationship between mean and SD of PEF. There was no significant difference between the fixed-dose and adjusted-dose groups (89.3 ± 7.6 and 90.2 ± 3.8, respectively), but in 2 patients in the fixed-dose group, mean PEFs were < 70% and were poorly controlled. No significant differences were observed between the fixed-dose and adjusted-dose groups in mean PEF, minimum/maximum PEF, PEF in the morning − PEF in the evening, and mean PEF for each asthma symptom by the Mann-Whitney U test.

**Table 2:** PEF analyses as a percentage of personal best PEF

	Total	Fixed-dose group	Adjusted-dose group
Mean (%)	89.8 ± 5.6	89.3 ± 7.6	90.2 ± 3.8
CV (standard deviation/mean)	0.045 ± 0.022	0.044 ± 0.022	0.047 ± 0.023
Minimum/Maximum	0.74 ± 0.13	0.75 ± 0.15	0.72 ± 0.12
Morning – evening (%)	− 1.03 ± 1.65	−1.23 ± 1.38	−0.88 ± 1.86
Mean PEF with symptoms (%):			
Cough	87.1 ± 6.8	85.4 ± 8.0	88.1 ± 6.2
Dyspnea	85.5 ± 7.9	82.6 ± 10.9	86.8 ± 6.4
Wheeze	83.6 ± 7.7	81.8 ± 11.6	84.2 ± 6.5
Mean PEF in each month (%):			
January	88.5 ± 6.2	87.5 ± 8.5	89.2 ± 4.3
February	89.0 ± 7.8	85.8 ± 11.1	90.7 ± 5.3
March	87.9 ± 8.2	85.6 ± 11.2	89.4 ± 5.6
April	88.9 ± 7.1	85.6 ± 10.7	90.5 ± 4.0
May	88.8 ± 5.5	88.0 ± 7.1	89.3 ± 4.5
June	89.5 ± 7.9	88.4 ± 10.3	90.2 ± 6.5
July	90.3 ± 7.4	87.7 ± 10.8	92.2 ± 3.1
August	90.5 ± 6.9	88.8 ± 10.0	91.7 ± 3.7
September	90.8 ± 6.5	90.2 ± 9.3	91.1 ± 3.8
October	90.1 ± 5.7	90.0 ± 7.3	90.2 ± 4.5
November	89.2 ± 6.7	89.5 ± 8.3	89.0 ± 5.6
December	88.3 ± 6.5	88.0 ± 8.3	88.5 ± 5.2

Mean ± SD

No significant differences between the fixed-dose and adjusted-dose groups

We calculated the mean PEF for each month (Table 2). There was no significant difference in PEF between the fixed-dose and adjusted-dose groups in all months by the Mann-Whitney U test. However, in the relationship by month for all patients, PEF in September was significantly higher than that in July, August, and December by the Wilcoxon signed-rank test (*p* < 0.05, *p* < 0.05, and *p* < 0.01, respectively); PEF in December was significantly lower than that in February, April, and November (all *p* < 0.05).

### Difference in ACT scores between the fixed-dose and adjusted-dose groups

We calculated the mean ACT score for each month (Table 3). ACT scores of the fixed-dose group were significantly higher than those of the adjusted-dose group in January, September, October, November, and December (*p* < 0.01, *p* < 0.05, *p* < 0.05, *p* < 0.05, and *p* < 0.01, respectively). In the relationship by month for all patients, ACT score was significantly lower in May than in July, August, September, October, November, and December (*p* < 0.05, *p* < 0.01, *p* < 0.05, *p* < 0.05, *p* < 0.01, and *p* < 0.05, respectively); ACT score was significantly lower in January than in November (*p* < 0.05) and in April than in July (*p* < 0.05).

**Table 3:** ACT score

	Total	Fixed-dose group	Adjusted-dose group
January**	22.7 ± 2.3	24.4 ± 0.5	21.5 ± 2.3
February	22.9 ± 3.0	24.4 ± 0.5	22.0 ± 3.5
March	22.4 ± 2.8	22.2 ± 2.9	22.6 ± 2.9
April	22.4 ± 2.9	21.5 ± 2.6	22.9 ± 3.1
May	21.8 ± 2.9	23.1 ± 1.2	20.9 ± 3.4
June	23.0 ± 1.9	23.4 ± 1.6	22.9 ± 2.1
July	23.3 ± 2.5	23.2 ± 2.5	23.3 ± 2.5
August	23.6 ± 2.6	24.2 ± 1.0	23.2 ± 3.2
September*	23.3 ± 1.8	24.2 ± 1.0	22.7 ± 2.0
October*	23.1 ± 2.8	24.3 ± 0.8	22.2 ± 3.4
November*	23.6 ± 1.4	24.3 ± 0.8	23.1 ± 1.5
December**	22.9 ± 2.8	24.4 ± 1.0	21.9 ± 3.2

Mean ± SD

*p < 0.05, **p < 0.01 for comparison between the fixed-dose and adjusted-dose groups

### Relationship between frequency of asthma symptoms and time to recovery from exacerbation of asthma in the adjusted group

Sixteen patients adjusted their BFC dose according to symptom severity. Their data were used for this analysis. In the severity of asthma based on medication, the frequencies of exacerbation of asthma were 0.20 ± 0.60 times per month in Step 1, 0.71 ± 0.69 times per month in Step 2, and 1.32 ± 0.34 times per month in Step 3, and a significant difference was observed between those in Step 1 and Step 3 (*p* < 0.01). However, no significant differences were observed between backgrounds, such as sex, age, atopy/non-atopy, and the initial number of eosinophils. The mean times to recovery from asthma exacerbation were 8.1 ± 6.0 days in Step 1, 3.9 ± 7.5 days in Step 2, and 3.5 ± 4.9 days in Step 3. The time to recovery from asthma exacerbation in Step 1 was significantly longer than that in Step 2 (*p* < 0.05). However, no significant differences were shown between backgrounds, such as sex, age, and atopy/non-atopy, except the initial number of eosinophils. That in patients with under 300/mm^3^ of eosinophils was significantly shorter than in those with over 300/mm^3^ of eosinophils (2.96 ± 6.3 days vs 5.62 ± 6.6 days, *p* < 0.05). Although patients in Step 1 presented with few exacerbations, recovery took a long time once they had exacerbation of asthma. However, PEF at BFC increase was not significantly different between Steps (Step 1:Step 2:Step3 = 84.4 ± 7.7%:89.2 ± 6.8%:84.7 ± 6.1%).

### Relationship between asthma symptoms and time of BFC dose increase

**Fig. 2:**
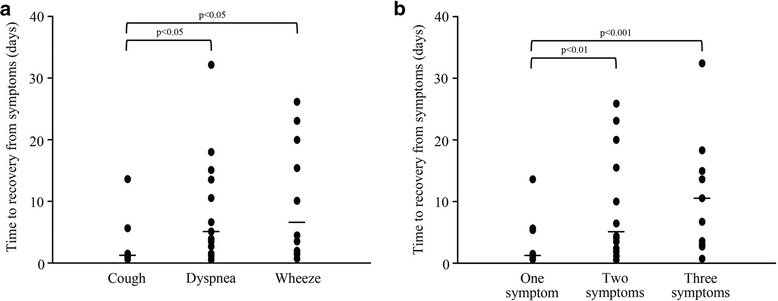
Relationship between types of asthma symptoms and time to recovery from asthma symptoms when BFC dose was increased (**a**). Relationship between number of asthma symptoms and time to recovery from asthma symptoms when BFC dose was increased (**b**). In A, the time to recovery from asthma symptoms was significantly shorter when patients increased the BFC dose in the presence of cough alone compared when they did so in the presence of dyspnea (*p* < 0.05) or wheeze (*p* < 0.05). There was no significant difference between dyspnea and wheeze. In B, the time to recovery from asthma symptoms was significantly shorter when patients increased the BFC dose in the presence of only one symptom compared with two (*p* < 0.01) or three (*p* < 0.001) symptoms. There was no significant difference in time to recovery between two and three symptoms

Mean PEF measured in the absence of any symptoms was determined for each patient. We listed the events at which BFC was increased because of asthma symptoms. Then, the period from the day when BFC was increased to the day when PEF was recorded in the absence of any symptoms was determined. The time to recovery from asthma symptoms was significantly shorter when patients increased the dose of BFC in the presence of only cough (18 events, 1.4 ± 3.3 days) compared with when they increased it in the presence of dyspnea (25 events, 5.3 ± 7.5 days, *p* < 0.05) or wheeze (17 events, 6.6 ± 8.9 days, *p* < 0.05; Fig. 2a). There was no significant difference between time to recovery in the presence of dyspnea and wheeze. The time to recovery from asthma symptoms was significantly shorter when patients increased the BFC dose in the presence of only one symptom (27 events, 1.3 ± 2.9 days) compared with two (23 events, 5.7 ± 7.8 days, *p* < 0.01) or three (10 events, 10.5 ± 9.6 days, *p* < 0.001) symptoms (Fig. 2b). There was no significant difference between the PEF recorded in the presence of two and three symptoms.

### Relationship between PEFs and time of BFC dose increase

**Fig. 3:**
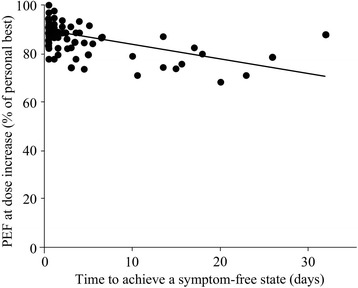
Relationship between PEFs and time to recovery from asthma symptoms with an increased BFC dose. The relationship is shown between PEF when BFC dose was increased (Y) and the time for the dose increase to achieve PEF greater than PEF in the absence of any symptoms (X), or when the BFC dose was increased without concomitant decrease in PEF, X was the days to decrease the BFC dose from day when the BFC dose was previously increased. The regression line was Y = − 0.591X + 89.2 (r^2^ = 0.299, *p* < 0.001)

We analyzed PEF when patients increased BFC dose, because patients sometime feel inexpressible asthma symptoms or asthma symptoms other than cough, dyspnea, or wheezing. We determined the events when BFC dose was increased because of asthma symptoms. Then, we calculated the time from the day when BFC was increased to the day when PEF at symptom-free status was achieved. Eighty-four events with 16 patients who adjusted their BFC dose were included in this analysis. PEF when patients increased BFC dose was 86.8 ± 6.9% of personal best PEF. Mean time to improve asthmatic symptoms, when the dose was increased to achieve a PEF greater than the PEF in the symptom-free state, was 2.0 ± 3.2 days. Fig. 3 shows the relationship between PEF when the dose was increased (Y) and days for the increased dose to achieve a PEF greater than PEF in the symptom-free state (X). When the BFC dose was increased without a concomitant decrease in PEF, X was the number of days required to decrease the BFC dose from the day the BFC dose was increased. The regression line was Y = − 0.591X + 89.2 (r^2^ = 0.299, *p* < 0.001). Because patients assessed their asthmatic status in the morning and evening, the shortest time to recover from asthmatic symptoms is half a day; for example, a patient presented with asthmatic symptoms in the morning and inhaled the adjusted dose of BFC, and the symptoms resolved by evening of the same day. The half-day value of “X = 0.5 days” represents the best timing for increasing the dose of BFC. When X is 0.5 days, PEF is 88.9% according to linear regression. To achieve this best timing, BFC should be increased before PEF is decreased to 88.9% of personal best PEF for all data. When BFC was decreased to 80% of personal best PEF, 15.6 days were required to recover from the asthma symptoms, as shown by the regression line.

## Discussion

Based on our results, to minimize the duration of asthma symptoms, BFC should be increased when the symptoms are mild. Our subjects showed high treatment adherence (94.9%) for over 8 months and therefore were good subjects for our study, even though the number of patients with mild asthma was small, because most of the patients in our university hospital have severe asthma. Furthermore, to include cases of clear asthma symptoms, we also enrolled patients with controlled asthma. When taking mean personal best PEF as 100%, 18/28 patients had PEF of > 90% and 26/28 patients had > 80%. Morning dipping was a decrease of only 1.03%. These subjects were therefore suitable to evaluate the relationship between BFC dose and PEF fluctuation, although some of them did not have asthma symptoms. In the study design, it is ideal to randomly separate adjusted and non-adjusted dose groups at the start. However, the ethics committee of our institution does not approve studies that prohibit adjustment of BFC because of patient rights. However, no significant difference was observed in background factors between these groups, except for sex, and these groups were suitable subjects for this study. Also, many previous publications analyzed the relationship between two different groups, such as SMART and non-SMART groups [4–16]. Yet, no study has evaluated the best timing of increasing of the BFC dose. In our study, we analyzed the data in the adjusted dose group and successfully determined the exact timing for increasing the dose of BFC.

In the PEF for each asthma symptom, the differences were very small, 87.1% in cough and 83.6% in wheeze, and the decrease from personal best PEF was small. In the Global Strategy for Asthma Management and Prevention, the dose of inhaled corticosteroid should be increased, if PEF has fallen by > 20% for more than 2 days [18]. However, our patients had wheezing at 83.6% of personal best PEF, and they increased the BFC dose when PEF had fallen to 86.8%. Indeed, some publications have reported that patients with mild asthma have higher sensitivity to dyspnea than those with severe asthma [20, 21]. Our study enrolled patients with mild and stable asthma, because the start and end in one asthmatic episode should be clear. Therefore, sensitivity to dyspnea might differ depending on the severity of asthma. Thus, to achieve symptom-free status, patients with mild or stable asthma should increase BFC when their PEF has fallen to 88.9%, not 80%. In severe asthma, to determine which is the best timing, 88.9% or 80%, we must distinguish between those groups who increased BFC dose when PEF had fallen to 88.9% or 80%. We are currently planning another study to investigate this.

Asthma symptoms tend to fluctuate seasonally, and thunderstorms and pollen are risk factors for exacerbation of asthma [22]. Generally, control of asthma is worse in spring and autumn, particularly in September [23]. In our study, seasonal factors also contributed the control of asthma. The ACT scores in autumn and winter were lower in the adjusted-dose group. Basal BFC dose was higher in the fixed-dose group than in the adjusted-dose group, but there was no significant difference. Differences in basal dose may contribute to these ACT scores. However, ACT scores in the adjusted-dose group could be improved by adjusting BFC at the appropriate time, according to our results. Some differences in PEF and ACT score between months were observed in tests of significance. But, a significant positive correlation between PEF and ACT scores was found between the months (ACT score = 0.375 X PEF (%) – 10.605, r^2^ = 0.431, *p* < 0.05). Therefore, there is no essential difference between ACT scores and PEF in each month. Good control of asthma was achieved by adjustment of BFC, and these regimens are very effective for long-term management of asthma.

The number of patients in the adjusted group was smaller than expected. Significant sex differences were found between the fixed-dose and adjusted-dose groups. Twelve patients did not adjust their BFC dose during the study period, although we had explained to them that they could adjust the dose for asthma symptoms. We asked patients in the fixed-dose group their reasons for not adjusting BFC doses, and they gave two main reasons: either it was not necessary to increase BFC because they did not have any asthma symptoms, which implies that their asthma was under control, or they could not determine when BFC should be increased, because they were not given detailed suggestions for increasing the dose by their physician. Also, the mean time to recovery was longer in patients with Step 1. The reason for this was that they could not determine the optimal timing for increasing the BFC dose, because they had less experience of exacerbations of asthma. Our results will be useful for those patients to help them determine when BFC should be increased, and these patients should be instructed in great detail.

With regard to SMART, we also asked the patients who did not use SMART about why they did not use BFC for symptom relief. Again, the reasons were twofold. The first was that BFC could not be used when at work. This is a very important problem in Japan to address. These patients may need sufficient regular doses of BFC more than SMART. We have previously reported that young adults without a primary care physician frequently visited our emergency department with asthma symptoms even though they probably needed only regular asthma treatment [24]. The second reason was that their symptoms were mild, they preferred to wait for the next scheduled dose and increase the dose then. According to our findings, the BFC dose should be increased even if asthma symptoms are mild. No studies have demonstrated the superiority of SMART compared with AMD, but SMART is theoretically better than AMD because SMART treats asthmatic symptoms immediately. Nonetheless, well controlled asthma could be maintained by AMD, before PEF decreases to 89.9% of personal best PEF.

Regarding differences in sex, 75% (12/16) of patients were female in the dose-adjusted group, and 100% (4/4) of patients treated with both AMD and SMART were female; there were more male than female subjects in the fixed-dose group. However, no significant differences were found in baseline characteristics and mean PEF between male and female subjects. Women with asthma are reported to be at low risk of poor adherence to inhaled corticosteroids [25]. Similarly, they have a significantly lower risk for non-adherence in the event of exacerbation [26]. Adherence to treatment improves when the patient wants to control their symptoms. Because treatment adherence among women is good, the women in our study had adjusted their BFC dose in accordance with our instructions.

In relation to concomitant drugs, 50% of patients were treated by BFC alone in the adjusted-dose group, compared with only 33% of patients in the fixed-dose group. We could not find any previous publications with similar findings. We could not identify the reason from the questionnaire responses in our study. One possibility may be that although our subjects had good adherence, adjusting the BFC dose may have been complicated for patients taking multiple medications. For such patients, full regular treatment is needed without the use of additional inhalers.

We think that PEF would be a more useful reference marker than symptoms for increasing BFC because PEF allows for objective evaluation of dyspnea and involves less error. Relying on symptom severity might not be an accurate enough method because patients with severe asthma show poor awareness of dyspnea [20, 21].

## Conclusion

To improve asthmatic symptoms in a shortest time for patients with mild or stable asthma, the BFC dose should be increased when PEF is decreased to 88.9% of the patient’s personal best PEF. When BFC dose is increased in the presence of cough as the only symptom, the asthma symptoms recover just 1.4 days after the dose increase. However, if the BFC dose is increased when PEF is decreased to 80% of the patient’s personal best PEF, it requires 15.6 days for the symptoms to completely subside. These criteria could help patients with mild or stable asthma to increase their BFC dose to achieve a quicker symptom-free status after asthma symptoms.

## Abbreviations

ACT: Asthma control test
AMD: Adjustable maintainable dose
BFC: Budesonide + formoterol
CV: Coefficient of variation
PEF: Peak expiratory flow
SABA: Short-acting β2-agonist
SD: Standard deviation
SMART: Symbicort maintenance and reliever therapy
